# Neuronal Mechanism for Compensation of Longitudinal Chromatic Aberration-Derived Algorithm

**DOI:** 10.3389/fbioe.2018.00012

**Published:** 2018-02-23

**Authors:** Yuval Barkan, Hedva Spitzer

**Affiliations:** ^1^Biomedical Engineering Department, Faculty of Engineering, Tel Aviv University, Tel Aviv, Israel; ^2^Electrical Engineering School, Faculty of Engineering, Tel-Aviv University, Tel-Aviv, Israel

**Keywords:** aberration, chromatic adaptation, compensatory mechanisms, computer model, visual perception

## Abstract

The human visual system faces many challenges, among them the need to overcome the imperfections of its optics, which degrade the retinal image. One of the most dominant limitations is longitudinal chromatic aberration (LCA), which causes short wavelengths (blue light) to be focused in front of the retina with consequent blurring of the retinal chromatic image. The perceived visual appearance, however, does not display such chromatic distortions. The intriguing question, therefore, is how the perceived visual appearance of a sharp and clear chromatic image is achieved despite the imperfections of the ocular optics. To address this issue, we propose a neural mechanism and computational model, based on the unique properties of the *S*-cone pathway. The model suggests that the visual system overcomes LCA through two known properties of the *S* channel: (1) omitting the contribution of the *S* channel from the high-spatial resolution pathway (utilizing only the *L* and *M* channels). (b) Having large and coextensive receptive fields that correspond to the small bistratified cells. Here, we use computational simulations of our model on real images to show how integrating these two basic principles can provide a significant compensation for LCA. Further support for the proposed neuronal mechanism is given by the ability of the model to predict an enigmatic visual phenomenon of large color shifts as part of the assimilation effect.

## Introduction

The human eye is affected by the imperfections of its optics, which degrade the quality of the retinal image and ultimately impose limits on vision. These imperfections have both spatial and chromatic implications. One of the most dominant chromatic implications is the phenomenon of longitudinal chromatic aberration (LCA). LCA is a significant and dominant attribute of the visual system and has been studied and measured extensively (e.g., Bedford and Wyszecki, 1957; Charman and Jennings, 1976).

**Figure 1:**
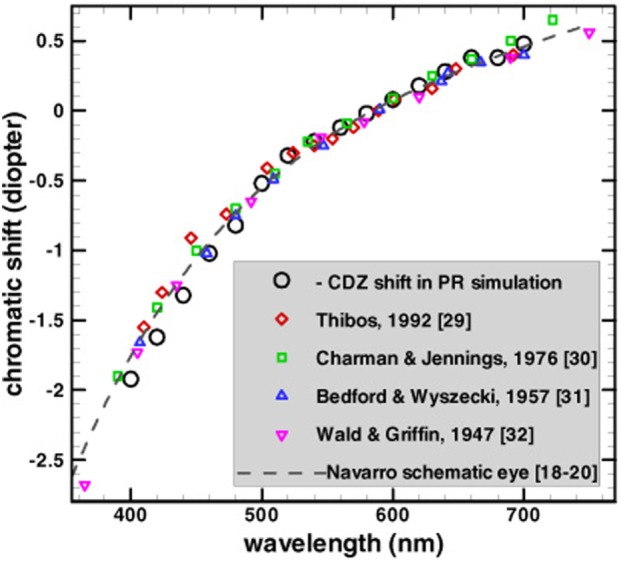
Comparison of refractive power (chromatic shift) reported by several studies. Note that the chromatic shift is much larger for the short wavelengths (blue photoreceptor) than for the long wavelengths (red photoreceptor). All the data are adjusted vertically to have a zero value at the reference wavelength of 589 nm giving the longitudinal chromatic aberration a refractive power of about two diopters. This image has been taken with permission from The Optical Society (Chen et al., 2003).

Longitudinal chromatic aberration is induced by the dependence of the refractive power of the lens on wavelength. As can be seen in Figure 1, the ocular refractive power is higher for shorter wavelengths (Bedford and Wyszecki, 1957). The accommodation mechanism of human eyes can determine the focus for each wavelength, but it is impossible to bring all of the wavelengths to focus simultaneously (Wandell, 1995). The phenomenon of LCA has been measured extensively, both by psychophysically (Wald and Griffin, 1947; Ivanoff, 1953; Bedford and Wyszecki, 1957; Jenkins, 1963; Howarth and Bradley, 1986) and retinoscopy methods (Charman and Jennings, 1976; Rynders et al., 1998). These studies showed that LCA has a refractive power of about two diopters (*D*), across the visible spectrum (Figure 1).

An alternative method of representing the chromatic aberration is through the modulation transfer function (MTF), which describes the sensitivity as a function of the spatial frequency and the wavelength. Due to the LCA, the MTF of the *S*-cone (blue) channel has a lower frequency cutoff (by a factor of 3–5) than the MTF of the *M*/*L* cone channels (red–green) (Shevell, 2003).

An additional factor that limits the visual acuity of the *S*-pathway is the low density of the *S* photoreceptors at the retinal mosaic. It is plausible that this low density has evolved in the visual system, in order not to have more sensors than the optical MTF can utilize. The MTF thus would be limited by both the LCA and photoreceptor density which, as mentioned above, are not independent factors. Calkins (2001) showed that the *S*-cone density can be a consequence of efficient Nyquist sampling: “…the eye’s optics together with what may be called ‘typical’ viewing conditions effectively limit any evolutionary pressure to pack S cones into the photoreceptor mosaic with a Nyquist rate greater than about 7–8 cycles deg^-1^.” If we approximate the *S* mosaic as triangular for ease of calculation, this sampling rate would correspond to an upper limit of foveal density in the human retina of 2,000–2,500 S cones mm^-2^. Various anatomical measurements of the distribution of *S* cones in the human retina, both direct and indirect, converge to a similar estimate: *S* cones peak in density at about 2,000 cells mm^-2^, just outside the center fovea, representing 5–10% of the cone population (Curcio et al., 1991).

The consequence of the LCA is that the retinal image will be focused only for the “green” wavelengths, and for the most part will be out of focus for the bluish wavelengths. The consequent image would be expected to have colored borders (“fringes”)—similar to that seen with a cheap lens (Valberg, 2005). Although it is not possible to remove these chromatic defects from a lens, an efficient optical system should be designed to minimize the distortion caused by the LCA. For example, it is possible to correct chromatic aberration through a combination of two or more lenses, in such a way that the aberration of each lens compensates for the aberration of the other lens (achromatic lens). In the human visual system, this solution is impractical since we are continuously changing the focal distance.

A recent proposal suggests that Müller glial cells may play a role in reducing the chromatic aberration due to the fact that peripheral light at larger tilt angles will be rejected more readily (Labin and Ribak, 2010). Another suggestion is that the short-wavelength absorbing pigments of the ocular media may have a function in limiting the chromatic aberration (Walls, 1963; Nussbaum et al., 1981). However, spectral filtering in the ocular media has a relatively small effect on the MTF (Shevell, 2003) and none of these optical features (Walls, 1963; Labin and Ribak, 2010) is sufficient to explain the lack of perceived distortion at sharp achromatic edges.

It is therefore intriguing to understand how notwithstanding the imperfections of the ocular optics, including the LCA, the perceived visual appearance is still a sharp and clear image. Since the optical system of the eye cannot apparently account for the correction, it is reasonable to suppose that the neuronal system acts to reduce the distortion (Shevell, 2003; Valberg, 2005). It should be appreciated that a non-optical system, such as the neuronal mechanism, cannot fully compensate for the optical limitations, since some of the physical information is lost. (This is exhibited by the limited MTF.)

Several studies have indeed suggested that there must be neural compensation for the eye’s aberrations. Although no specific mechanism has been described (Hay et al., 1963; Artal et al., 2004), a number of compensatory options have been suggested, most of which are related to the McCollough effect (ME) (Hay et al., 1963; Broerse et al., 1999; Grossberg et al., 2002). The ME is a long-term after-effect that can last from hours up to 3 months (Jones and Holding, 1975).

The rationale to associate the ME with the LCA phenomenon derives mainly from its long-lasting temporal property, and its relation to chromatic edges (McCollough, 1965). The proposed compensatory models are composed of oriented receptive fields (RFs) (multiplexed simple cells) consisting of both chromatic- and achromatic-separated subunits (Broerse et al., 1999; Grossberg et al., 2002). The elimination of the chromatic distortion is then explained by invoking a learning mechanism that inhibits the appearance of chromatic edges adjacent to achromatic edges.

These models have been supported by experiments that demonstrate that there is a long-term adaptation to chromatic aberration caused by a wedge prism. It has been demonstrated that dispersion of light passing through a wedge prism produces bluish and yellowish fringes on achromatic edges. These perceived fringes disappear when the prisms are worn for a long period of time (about 2 days) (Hay et al., 1963). This adaptation of the visual system supports the existence of a long-term corrective neural compensation mechanism.

These models can be accounted for neuronal compensation only when the chromatic aberration refractive power is constant. However, the refractive power of the LCA constantly changes due to the pupil size (that is determined by the amount of light and the accommodation of the eye). The temporal scale of pupil size change is within the range of 200–500 ms, which is faster by orders of magnitude than the neuronal adaptation mechanisms described above (which can last hours to months). Consequently, there is necessity for an additional mechanism that compensates for chromatic aberration and is less dependent on a momentary magnitude of chromatic aberration.

This means that a neural mechanism that compensates for general LCA phenomenon still remains to be discovered. If such a neural mechanism exists, it is expected that not only will it have the ability to compensate for the LCA phenomenon but will also be able to predict the visual phenomena generated by the compensation neuronal mechanism.

In this paper, we propose a plausible computational model of the retina that can compensate for LCA. The model is based on well-known retinal color-coding RFs and does not require a learning process. The validity of the suggested model is supported by its ability to predict related visual phenomena.

## Model

The model computes the perceived color in accordance with the response of retinal color-coding ganglion cells (Daw, 2012). This calculation involves two main stages. The first stage evaluates the response ganglion cells of type I (*L*/*M* and *M*/*L*, on center cells) and type II (*S*/*LM*, on coextensive cells). This stage includes the calculation of the RF response of each color-coding cell that also exhibits a remote adaptation mechanism. In addition, this stage also includes two separated pathways related to the luminance and chromatic knowledge of the two cell types. The second stage of the model proposes a novel transformation of the ganglion cell response into a perceived image by using an inverse function. The source code for the model simulation is available at https://github.com/yubarkan/LCAcompensation/.

### Response of the Opponent RF

The retinal ganglion cells receive their input from the cones through several chemical and electrical processing layers (Shevell, 2003). The retinal ganglion cells then perform an adaptation of the first order. The adaptation of the first order is modeled here through adaptation of the cell inputs, rather than adaptation of the RF subregions (Spitzer and Semo, 2002; Spitzer and Barkan, 2005). We therefore define the adapted ganglion cell input signals as follows:

(1) $$\begin{array}{ll} L_{\text{pr\_adapted}} & =\frac{L_{\text{photo}-r}}{L_{\text{photo}-r}+\sigma_{L}\;\left(L_{\text{photo}-r}+L_{\text{remote}}\right)},\\ M_{\text{pr\_adapted}} & =\frac{M_{\text{photo}-r}}{M_{\text{photo}-r}+\sigma_{M}\;\left(M_{\text{photo}-r}+M_{\text{remote}}\right)},\\ S_{\text{pr\_adapted}} & =\frac{S_{\text{photo}-r}}{S_{\text{photo}-r}+\sigma_{S}\;\left(S_{\text{photo}-r}+S_{\text{remote}}\right)}, \end{array}$$

where *L*_adapted_, *M*_adapted_, and *S*_adapted_ are the adapted inputs from the cones and σ*_L,M,S_* are remote and local adaptation signals and are defined as

(2) $$\begin{array}{ll} \sigma_{L} & =a\cdot L_{\text{photo}-r}+b+c\cdot L_{\text{remote}},\\ \sigma_{L} & =a\cdot M_{\text{photo}-r}+b+c\cdot M_{\text{remote}},\\ \sigma_{S} & =a\cdot S_{\text{photo}-r}+b+c\cdot S_{\text{remote}}, \end{array}$$

where the remote signals are defined as

(3) $$\begin{array}{ll} L_{\text{remote}}\left(x,y\right) & \;=\;\underset{\text{cen}-\text{area}}{\iint }L_{\text{photo}-r}\left(x^{\prime },y^{\prime }\right)\cdot f_{\text{remote}}\left(x-x^{\prime },y-y^{\prime }\right)\cdot {\mathrm{dx}}^{\prime }\cdot {\mathrm{dy}}^{\prime },\\ M_{\text{remote}}\left(x,y\right) & \;=\;\underset{\text{cen}-\text{area}}{\iint }M_{\text{photo}-r}\left(x^{\prime },y^{\prime }\right)\cdot f_{\text{remote}}\left(x-x^{\prime },y-y^{\prime }\right)\cdot {\mathrm{dx}}^{\prime }\cdot {\mathrm{dy}}^{\prime },\\ S_{\text{remote}}\left(x,y\right) & \;=\;\underset{\text{cen}-\text{area}}{\iint }S_{\text{photo}-r}\left(x^{\prime },y^{\prime }\right)\cdot f_{\text{remote}}\left(x-x^{\prime },y-y^{\prime }\right)\cdot {\mathrm{dx}}^{\prime }\cdot {\mathrm{dy}}^{\prime }. \end{array}$$

The “remote” area is composed of an annulus-like shape around the entire RF region (Spitzer and Barkan, 2005). Its weight function (*f*_remote_) is modeled as a decaying exponent at the remote area as follows:

(4) $$f_{\text{remote}}\left(x,y\right)=\frac{1}{\pi \cdot \rho_{\text{remote}}}\text{exp}\;\left(-\frac{x^{2}+y^{2}}{{\rho_{\text{remote}}}^{2}}\right)\;;x,y\in \text{remote\_area.}$$

The spatial response profile of the two subregions of the retinal ganglion RF, “center” and “surround,” is expressed by the known difference-of-Gaussians (DOG). It should be noted that the calculation of the DOG is performed on the adapted inputs.

The “center” signals of the two spectral regions, *L*_cen_, *M*_cen_, are defined as integrals of the adapted inputs (*L*_adapted_, *M*_adapted_; Eq. 1) over the center subregion, with a Gaussian decaying spatial weight function (*f_c_*):

(5) $$\begin{array}{ll} L_{\text{cen}}\left(x,y\right) & =\underset{\text{cen}-\text{area}}{\iint }L_{\text{pr\_adapted}}\left(x^{\prime },y^{\prime }\right)\cdot f_{c}\left(x-x^{\prime },y-y^{\prime }\right)\cdot {\mathrm{dx}}^{\prime }\cdot {\mathrm{dy}}^{\prime },\\ M_{\text{cen}}\left(x,y\right) & =\underset{\text{cen}-\text{area}}{\iint }M_{\text{pr\_adapted}}\left(x^{\prime },y^{\prime }\right)\cdot f_{c}\left(x-x^{\prime },y-y^{\prime }\right)\cdot {\mathrm{dx}}^{\prime }\cdot {\mathrm{dy}}^{\prime }, \end{array}$$

while *L*_cen_(*x*,*y*) at each location represents the subregion response of the center area, which is centered at location *x*, *y*, …*f_c_* and is defined as

(6) $$f_{c}\left(x,y\right)=\frac{1}{\pi \cdot \rho_{\text{cen}}}\text{exp}\;\left(-\frac{x^{2}+y^{2}}{{\rho_{\text{cen}}}^{2}}\right)\;;x,y\in \text{center\_area},$$

where ρ represents the radius of the center region of the RF. The “Surround” signals are defined in the same manner as follows (with a spatial weight function three times larger than that of the “center”):

(7) $$\begin{array}{ll} L_{\text{sur}}\left(x,y\right) & =\underset{\text{sur}-\text{area}}{\iint }M_{\text{pr\_adapted}}\left(x^{\prime },y^{\prime }\right)\cdot f_{s}\left(x-x^{\prime },y-y^{\prime }\right)\cdot {\mathrm{dx}}^{\prime }\cdot {\mathrm{dy}}^{\prime },\\ M_{\text{sur}}\left(x,y\right) & =\underset{\text{sur}-\text{area}}{\iint }L_{\text{pr\_adapted}}\left(x^{\prime },y^{\prime }\right)\cdot f_{s}\left(x-x^{\prime },y-y^{\prime }\right)\cdot {\mathrm{dx}}^{\prime }\cdot {\mathrm{dy}}^{\prime }, \end{array}$$

where *f_s_* is defined as a decaying Gaussian over the surround region:

(8) $$f_{s}\left(x,y\right)=\frac{1}{\pi \cdot \rho_{\text{sur}}}\text{exp}\;\left(-\frac{x^{2}+y^{2}}{{\rho_{\mathrm{sur}}}^{2}}\right)\;;x,y\in \text{surround\_area}.$$

The total weight of *f_c_* and *f_s_* is 1.

The response of the cells is expressed by the subtraction of the center and surround-adapted responses as follows:

(9) $$\begin{array}{ll} L^{+}M^{-}\left(x,y\right) & =L_{\text{cen}}^{\;}\left(x,y\right)-M_{\text{sur}}^{\;}\left(x,y\right),\\ M^{+}L^{-}\left(x,y\right) & =M_{\text{cen}}^{\;}\left(x,y\right)-L_{\text{sur}}^{\;}\left(x,y\right). \end{array}$$

The *S*/*LM* retinal color-coding cell is known as the small bistratified ganglion cell. The RF of this cell is known in the literature to be coextensive (type II), i.e., it has mainly chromatic opponency rather than spatial opponency (Hubel and Wiesel, 1968; de Monasterio, 1978; Derrington et al., 1984). Accordingly, the response of the *S*-cone opponent is modeled here as a type-II RF. The *S*/*LM* signal was therefore modeled through integration of the chromatic difference (*S*/*LM*) over the whole RF of this cell type:

(10) $$\begin{array}{ll} S^{+}LM^{-}\left(x,y\right) & =\underset{\text{blue}-\text{RF}-\text{area}}{\iint }\left[S_{\text{adapted}}\left(x^{\prime },y^{\prime }\right)-\frac{L_{\text{adapted}}\left(x^{\prime },y^{\prime }\right)+M_{\text{adapted}}\left(x^{\prime },y^{\prime }\right)}{2}\right]\\ & \cdot f_{s\text{\_center}}\left(x-x^{\prime },y-y^{\prime }\right)\cdot {\mathrm{dx}}^{\prime }\cdot {\mathrm{dy}}^{\prime }. \end{array}$$

The spatial weight function of the RF, *f_c__*_center_, is defined as in Eq. 7.

### Transformation to Image

The purpose of this stage is to model how the visual system transforms the RF responses to a perceived image. We suggest that in order to eliminate the effect of the blurred *S*/*LM* channel, the visual system has to very precisely exclude this channel from the processing of the high-spatial resolution channel. This suggestion is in accordance with the consensus in the literature and with accumulated evidence indicating that the chromatic information that includes the *S*/*LM* information is processed through a unique pathway, i.e., the koniocellular pathway (Hendry and Reid, 2000). Additional support for our proposal is derived from the observation that the *L* and *M* data that code high-spatial resolution information are processed independently through the parvocellular pathway (Livingstone and Hubel, 1988; Van Essen and Gallant, 1994; Hendry and Reid, 2000; Sincich and Horton, 2005).

In order to perform a transformation from the opponent signals [*L* + *M*−, *M* + *L*−, and *S* + (*L* +* M*)−] to perceived triplet *LMS* values, we propose a functional minimization framework. We imply that the perceived values should satisfy the following equations:

(11) $$\begin{array}{ll} L^{+}M_{\;}^{-} & =L_{\text{per}}-M_{\text{surround\_per}},\\ M^{+}L_{\;}^{-} & =M_{\text{per}}-L_{\text{surround\_per}}. \end{array}$$

*L*_surround_per_ and *M*_surround_per_ are defined in Eq. 7, but here they are related to the perceived domain rather than adapted input signals. We define the following error function:

(12) $$\begin{array}{l} E\left(L_{\text{per}},M_{\text{per}}\right)={\left[L_{\text{per}}-\left(L^{+}M_{\;}^{-}+M_{\text{surround\_per}}\right)\right]}^{2}+{\left[M_{\text{per}}-\left(M^{+}L_{\;}^{-}+L_{\text{surround\_per}}\right)\right]}^{2}. \end{array}$$

This function is the square error between the estimation of *L*_per_, *M*_per_, and the satisfaction of Eq. 12. This error function can be minimized by various methods. For simplicity, we show the implication of the gradient descend method as follows (Snyman, 2005):

(13) $$\begin{array}{ll} \frac{\partial L_{\text{per}}}{\mathrm{\partial t}} & =-\frac{\mathrm{\partial E}\left(L_{\text{per}},M_{\text{per}}\right)}{\partial L_{\text{per}}},\\ \frac{\partial M_{\text{per}}}{\mathrm{\partial t}} & =-\frac{\mathrm{\partial E}\left(L_{\text{per}},M_{\text{per}}\right)}{\partial M_{\text{per}}}. \end{array}$$

Thus, we obtain the following iterative equations:

(14) $$\begin{array}{l} {L^{i}}_{\text{per}}\;=\;{L^{i-1}}_{\text{per}}+\mathrm{dt}\cdot \;\left[2\cdot \;\left({L^{i-1}}_{\text{per}}-L^{+}M_{\;}^{-}-{M^{i-1}}_{\text{surround\_per}}\right)\right.\left.+\;2\cdot \mathrm{fs}\left(0,0\right)\cdot \;\left({M^{i-1}}_{\text{per}}-M^{+}L_{\;}^{-}-{L^{i-1}}_{\text{surround\_per}}\right)\right]\;\;,\\ {M^{i}}_{\text{per}}\;=\;{M^{i-1}}_{\text{per}}+\mathrm{dt}\cdot \;\left[2\cdot \;\left({M^{i-1}}_{\text{per}}-M^{+}L_{\;}^{-}-{L^{i-1}}_{\text{surround\_per}}\right)\right.\left.+\;2\cdot \mathrm{fs}\left(0,0\right)\cdot \;\left({L^{i-1}}_{\text{per}}-L^{+}M_{\;}^{-}-{M^{i-1}}_{\text{surround\_per}}\right)\right]. \end{array}$$

This iteration process provides the perceived *L* and *M* values, independently of the *S*/*LM* channel (see the rationale above).

The perceived *S*-channel value (*S*_per_) is calculated after evaluating the *L* and *M* perceived values (Eq. 14) by using the following equation:

(15) $$S_{\text{per}}=S^{+}{\left(L+M\right)}_{\;}^{-}+\left(L_{\text{per}}+M_{\text{per}}\right)∕2.$$

According to our model, the *S*_per_ contributes to the perceived color and not to the perceived luminance. Thus, the perceived brightness is expressed solely by the *L* and *M* values.

## Methods

In this section, we describe the different tools and parameters used in the model simulation. The same sets of parameters were used for all the simulated images that are presented in Section “Results.”

### Modeling Human Optics

**Figure 2:**
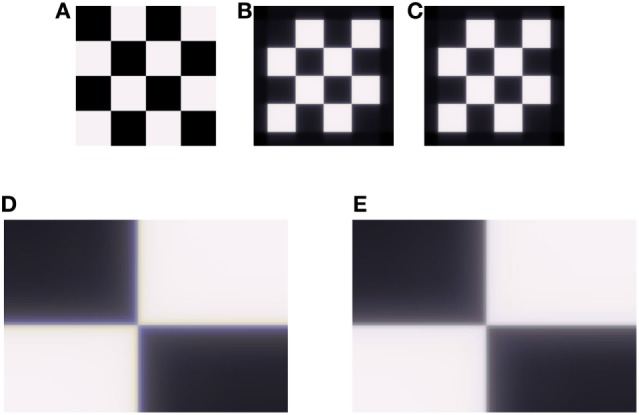
Demonstration of the longitudinal chromatic aberration (LCA) model on an achromatic grid **(A)**. **(B)** Retinal image, simulated by ISETBIO toolbox (see Methods). **(C)** Model prediction for perceived image. **(D)** Enlarged section of the retinal image **(B)**, the LCA can be seen in vicinity of the edges as lines of a blue–yellow color. **(E)** Enlarged section of model prediction **(C)**, where there is a correction of the chromatic distortion.

In order to evaluate the ability of our model to compensate for chromatic aberration, it is necessary to simulate the results from human optics on test images. We have used the Image System Engineering Toolbox for Biology ISETBIO,^1^ which provides a unique ability to simulate human optics in a real scene. For this purpose, we have used high-resolution, high-dynamic, multispectral image (HDRS) taken from the ISET High-Dynamic Range Multispectral Scene Database available by the Image Evaluation Tools.^2^ ISETBIO also includes the WavefrontOptics code developed by David Brainard, Heidi Hofer, and Brian Wandell. Their code implements methods to model human eyes by taking adaptive optics data from wave-front sensors and calculating the optical blur as a function of the wavelength. The toolbox relies on data collected by Thibos et al. We have chosen an illumination of blackbody at 6,500 K and uses WavefrontOptics to simulate the retinal image produced by human optics. Figure 2 is produced by this method.

### Response of the Opponent RF

In the first stage of the model, the adapted signals are calculated (Eqs. 1–4). The remote area was simulated as an annulus with a diameter of 35 pixels. The adaptation parameters were chosen as follows: *a* = 1, *c* = 1, representing equal strength for the local and remote adaptations (Eq. 4). The parameter “*b*,” which determines the strength of adaptation (Dahari and Spitzer, 1996; Spitzer and Barkan, 2005), was taken as *b* = 3.

The calculation of surround signals (Eq. 7) was calculated with fs (Eq. 8) having a decay constant (ρ) of 3 pixels. The response of the RFs was obtained by subtracting the center and surround-adapted responses (Eq. 9).

### Transformation to Image (Inverse Function)

The purpose of this section is to perform a transformation from the RF responses to a perceived image. The transformation was performed using the Jacobi iterative method (Eq. 14). The iteration process was initiated (*i* = 0) by assuming achromatic stimuli. Specifically, all channels were initiated with the following values:

$$L_{\text{per}}^{0}=M_{\text{per}}^{0}=S_{\text{per}}^{0}=\frac{L_{\text{adapted}}+M_{\text{adapted}}}{2}.$$

The iterative process converges to the predicted perceived image, while the color “fills-in” the stimulus.

## Result

The ability of the model to reduce the effect of LCA was tested on both the artificial and natural images. Retinal images were simulated by using the ISETBIO toolbox, which takes into account the properties of the human optical system (see Methods). The LCA effect is very prominent when zooming into areas of luminance or chromatic edges (Figure 2).

Figure 2 demonstrates the model’s performance on an artificial achromatic grid (Figure 2A) composed of equal energy squares. The image that is cast on the retina was calculated using ISETBIO (Figure 2B). It can be seen that this image (which simulates the eye’s optics, including the LCA) has major chromatic distortions adjacent to the borders (Figures 2B,D). The distortion appears “yellowish” (lack of blue) on the bright side of the border and “bluish” on the darker side. Figures 2C,E present the effect of the model, which simulates the retinal response and its perceived image. Figures 2B–E show that the model succeeds in significantly reducing the chromatic-border distortion.

**Figure 3:**
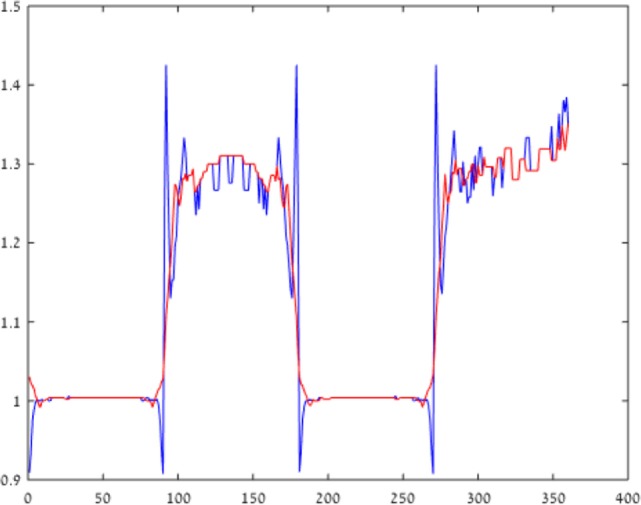
Blue–yellow chromatic contrast. Blue–yellow chromatic contrast at a cross-section of the retinal image is presented, across a horizontal line in Figure 2 (in blue), while the model correction for longitudinal chromatic aberration (LCA) is represented by the red line. It can be seen that the LCA, which is represented by the blue spikes, is reduced significantly by the model correction (red line), which eliminates the chromatic distortion.

Figure 3 plots the chromatic contrast, defined as the ratio between the value of the blue and yellow channels [B/(R + G)], across the *x*-axis of Figures 2B,C. This chromatic contrast represents the chromatic deviation from neutral hue (achromatic region). An achromatic region is characterized by a contrast value of 1, while the higher and lower values represent deviations toward bluish and yellowish chroma, respectively.

The blue curve plots the chromatic contrast across the cast image (Figure 2). The fringes of the plot are indicated by the large negative and positive spikes next to the borders (*x* = 90). The results given by our model (red line) show a significant reduction of the spike magnitude, indicating a significant reduction of the chromatic fringes. The deviation from white is also significantly diminished. It should be noted that there is some constant hue generated mainly on the “black” squares, which is a side effect of the ISETBIO simulation, rather than an ideal achromatic appearance (contrast value of 1).

**Figure 4:**
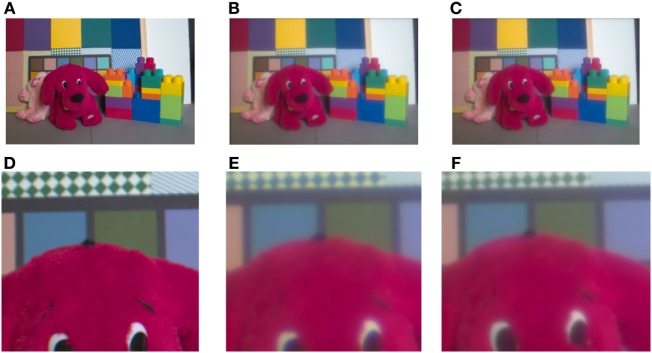
Demonstration of the longitudinal chromatic aberration (LCA) model. Demonstration of the model performance on the toys’ image **(A)** provided by Brian Wandell. **(B)** Retinal image, simulated by ISETBIO toolbox (see Methods). **(C)** Model prediction of the perceived image. **(D)** Enlarged section of retinal image (the LCA) can be seen in the vicinity of the edges as blue–yellow colored lines. **(D,E,F)** represent a magnified image of the puppy’s eyes and the chromatic pattern zone in the background of the images **(A,B)**, and model prediction **(C)**. The correction can be observed only after enlargement **(F)**. The bluish color, a manifestation of the chromatic aberration, is prominent in **(E)** and the model’s correction is seen clearly in **(F)**. The change in the bluish chroma is also clear in the background pattern in **(E)** and the greenish restoration in **(F)**.

We also tested the model’s ability to compensate for LCA on real images (Figure 4A), taken from the ISETBIO HDRS library. The optics of the eye was simulated using the ISETBIO (Figure 4B; see Methods). The results show that the model succeeds in correcting the chromatic distortions around borders (Figure 4C). The correction is prominent in the distorted puppy dog’s eye color and the distorted green–white pattern behind the dog (Figure 4D-F). Although the model significantly reduces the distortion caused by LCA, it can also cause some minor chromatic artifacts.

**Figure 5:**
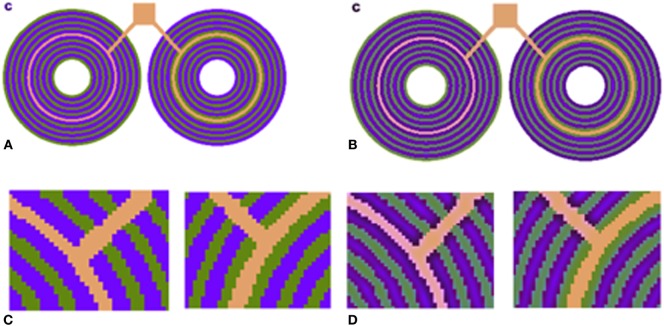
**(A)** *S*-cone pattern reported by Shevell and Monnier (2005). The pink and orange rings are actually physically identical. **(B)** Our model prediction. **(C)** Zoom-in version of **(A)** shows that the central ring is identical. **(D)** Zoom-in version of **(B)** shows that the model successfully predicts the chromatic shift, and the left-hand side ring appears pinkish.

The neuronal mechanism that we propose as capable of correcting for chromatic aberration is bound by the limitations of the spatial frequency of the *S*/*LM* channel (Eq. 10; see Model). In other words, a crucial part of the model suggests that the *S*/*LM* channel is processed through a spatial low-pass filter. If such a mechanism actually exists, we would predict that it would lead to visual phenomena that are prominent at stimuli with high frequencies of blue/yellow chromaticity. We would expect to see these phenomena as a blue–yellow assimilation effect, at high-spatial frequencies or among adjacent chromatic regions with sharp edges. These characteristics correspond closely to with a recent outstanding chromatic illusion, which is termed as “Chromatic induction from *S*-cone patterns” and described by Monnier and Shevell (2004) (Figure 5).

This illusion describes the perception of a chromatic specific narrow ring with color that differs completely, depending on the specific chromaticity of an adjacent ring (Figure 5). Psychophysical methods of analysis indicate that the chromatic shift is not directly dependent on the absolute blue channel intensity (*S*) of the blue component of the adjacent rings but rather on the relative amount of “blue” and “yellow” intensities (*S*/*LM*) in the adjacent rings (Shevell and Monnier, 2006).

We also tested our model on *S*-cone pattern stimuli, which have been reported by Monnier and Shevell (2004) to demonstrate prominent chromatic induction. The results (Figure 5) show that our model succeeds in predicting the trend of the perceived chromaticity shift toward the chromaticity of the adjacent ring (Figure 5D). The predicted chromatic shifts, between the two test chromaticities (the orange and pink rings) in terms of chromatic contrast [*S*/(*L* + *M*)], are about 0.31. This shift agrees with the perceived colors as measured psychophysically by Shevell and Monnier.

## Discussion

This manuscript describes a neuronal mechanism and a computational model, based on retinal chromatic RFs and visual pathways, that compensate for LCA. The model can significantly reduce the chromatic distortion at both the artificial and natural images (Figures 2 and 3). The proposal is supported by the observation that an artifact of chromatic assimilation, which is a predicted consequence of the model, corresponds to a well-known chromatic assimilation phenomenon described previously (Shevell and Monnier, 2005).

The model is based on the specific spatial and chromatic structure of the blue–yellow channel (*S*/*L* + *M*) RFs, which are spatially coextensive “type-II” small bistratified cell (SBC) (see Model; Hubel and Wiesel, 1968; de Monasterio, 1978; Derrington et al., 1984; Tailby et al., 2008; Crook et al., 2009; Martin and Lee, 2014) and correspond to the activities of the SBCs. These type-II RFs are incorporated into a retinal adaptation model (Spitzer and Barkan, 2005), and then the RF responses are subjected to an inverse function that mediates a transformation to perceived values. This transformation enables an evaluation of the model by consideration of an image domain, rather than merely on the basis of the RF responses.

There has been some dispute in the literature regarding the spatial coextensive nature of the SBC. The coextensive nature of the SBC has been described by many electrophysiological researchers (Hubel and Wiesel, 1968; de Monasterio, 1978; Derrington et al., 1984). A recent experiment reported that the SBC RF may not be spatially coextensive (Field et al., 2007). However, these results have been criticized first because the data in Field et al. (2007) were collected in the far retinal periphery (30–75° eccentricity), where more recent and broad reports of the RF were recorded within the central 20°(Hubel and Wiesel, 1968; de Monasterio, 1978; Derrington et al., 1984). Crook et al. (2009) found that the *S*-ON and *LM*-OFF responses were spatially coextensive, or nearly so. Furthermore, this trend of results was supported by large previous papers including recent reports and a review (Tailby et al., 2008; Crook et al., 2009; Martin and Lee, 2014).

A logical conclusion may be that the development of visual system has been strongly influenced by the natural visual scenery. Most of the sun’s spectral energy on earth is yellowish (550 nm) (Figure 1.2.1 in Wyszecki and Stiles, 1982), giving fewer chromatic edges in natural scenes than achromatic edges, and with a predominance of red–green chromatic edges over blue–yellow (Hansen and Gegenfurtner, 2009). The peak of the spectral luminance efficiency of the visual system (Wyszecki and Stiles, 1982) is similar to the peak of the sun’s spectral energy with the ocular lens tuned for optimal focus at the same wavelength. The chromatic aberration occurs in the short wavelengths, where there is both less solar irradiance and fewer chromatic edges in natural images. It therefore appears that the ocular lens is designed to provide the optimal performance at the prominent natural wavelength (~550 nm) while allowing the aberration at shorter wavelengths, which are less significant both for spatial and luminance information.

Although the ocular lens is tuned to the most “important wavelengths,” it still suffers from the consequences of the chromatic aberration. It is plausible that the neural system compensates for some of these optical imperfections (Wandell, 1995). We propose that the visual mechanism utilizes the absence of sharp blue–yellow edges to diminish the effect of chromatic distortions. In the model, this is replicated by the following mechanisms, whose existence is supported by psychophysics and neurophysiologic findings.

Luminance and high-spatial resolution chromatic information, under photopic light conditions, is obtained mainly from the *L* and *M* channels—which suffer less from LCA. This idea is supported by psychophysical evidence showing that the contribution of the *S* cone to luminance perception is negligible or null (Eisner and MacLeod, 1980; Wyszecki and Stiles, 1982). This knowledge has been also applied in the definition of the classical CIE color space where, for example, the *V* (λ)*s* describing the spectral luminance efficiency (i.e., perceived brightness vs. wavelength) come mainly from greenish and red light (Wyszecki and Stiles, 1982). As a result, brightness is calculated by perceived *L* and *M* values with almost no input from the *S* channel (Eq. 14), while the calculation of the chromaticity takes the contribution of the *S* value into account as well as the contribution of the other chromatic channels (Eq. 15).

The opponent RF structure of the *S* channels (SBCs) is both spatially coextensive and chromatically complementary (Dacey, 1996; Rodieck, 1998; Eq. 10). Such an RF blurs the blue–yellow information, so that their chromatic mixture yields an achromatic color. In addition, the spatio-chromatic structure [of *S*/(*L* + *M*) RF] yields a null response to achromatic edges, also in the presence of LCA affecting the *S* channel. In this way, the unique spatio-chromatic property minimizes the chromatic distortion (see Results; Figure 2).

In order to maintain the compensatory advantage at the retinal stage, which separates high-spatial frequency information from low-spatial frequency chromatic information, the system has to further process these two channels separately. There are physiological findings, which show that the SBC RF (with B/Y chromatic structure) indeed feeds a distinct chromatic pathway, i.e., the koniocellular pathway (Hendry and Reid, 2000). The origin of the koniocellular pathway lies in the SBC in the retina, and the pathway is then relayed by the koniocellular layer in the LGN to the cytochrome-oxidase blobs in V1. Several studies have reported that information on color *per se* and information on form are separated (Livingstone and Hubel, 1988; Van Essen and Gallant, 1994; Sincich and Horton, 2005). The information on form is derived solely from the parvocellular pathway [which lacks the *S*/(*L* + *M*) information]. The information on color, however, comes from both the koniocellular and parvocellular pathways. The parvocellular pathway sends inputs from layer 4*c*β to the blobs in layer 2/3, area V1. The two separate pathways (color and form) do have different anatomical inputs in the V2 area. Here, the thin stripes that code the color information are fed both from the konio and parvo pathways, whereas the pale strips, which code the form information, are fed only by the parvo pathway. The “form” pathway is therefore not affected by the deficiencies of the *S*/(*L* + *M*) pathway. Both pathways project to area V4 and additional higher visual areas.

Previous studies that proposed neuronal mechanisms to compensate for chromatic aberration (Hay et al., 1963; Broerse et al., 1999; Grossberg et al., 2002; Vladusich and Broerse, 2002) related these mechanisms to long-term after-effects, such as the ME—a long-term orientation-contingent color after-effect (McCollough, 1965). Vladusich and Broerse (2002) proposed a learning neuronal model that inhibits the fringes at luminance boundaries (caused by chromatic aberrations). Grossberg et al. (2002) proposed a learning mechanism whose primary function is to adaptively align the representations of the boundaries and surfaces, which are shifted due to the process of binocular fusion. Their mechanism was able to predict the ME. Since the ME has been previously suggested as the compensation mechanism for chromatic aberration, the model presented by Grossberg et al. (2002) was also regarded as a compensation model for LCA.

In our opinion, there are two main arguments against the idea that ME models can completely explain neuronal compensation to LCA. The first limitation of the above models (Broerse et al., 1999; Grossberg et al., 2002; Vladusich and Broerse, 2002) is that they assume that the magnitude of LCA effect depends solely on the magnitude of the luminance edge. However, the LCA effect also depends on additional optical factors, such as the pupil aperture (DeValois and DeValois, 1991), whose size changes dynamically in response to the level of ambient illumination and accommodation. Such learning mechanisms, therefore, would be expected to yield chromatic artifacts when the pupil aperture size changes and would therefore require continuous adaptation of the learning mechanism. The learning models described above may therefore be more applicable to transverse chromatic aberration (TCA), which does not depend on the pupil size. Thus, there could be two different and complementary mechanisms for the two types of aberrations, i.e., TCA and LCA.

An additional limitation of previous models (Broerse et al., 1999; Grossberg et al., 2002; Vladusich and Broerse, 2002) is their assumption that the LCA is triggered only by achromatic boundaries. In fact, chromatic aberration (and specifically the LCA) also occurs at iso-luminance chromatic boundaries, where there are no achromatic boundaries (Figure 1). Consequently, the above models fail to explain how the visual system processes chromatic fringes at non-achromatic borders.

The two types or mechanisms, the current proposed retinal model, and the above learning mechanisms can be synergetic in the visual system. The retinal mechanism performs an early-stage correction that eliminates most of the LCA effects, regardless of the degree of illumination and eye accommodation. The cortical learning mechanism (Watanabe et al., 1992; Broerse et al., 1999; Grossberg et al., 2002; Vladusich and Broerse, 2002; Grossberg, 2003) performs long-term adaptation that can adapt to specific ocular changes (such as lens defects that can be caused by aging or physical damage, etc.).

Although several studies have examined the improvement of visual acuity through optical correction of LCA (Campbell and Gubisch, 1967; Yoon and Williams, 2002; Artal et al., 2010), none found better than minor improvement (or none) of the contrast sensitivity. One may argue that these results suggest that LCA is not a real problem of the optical system, since correcting it does not create any significant improvement. However, in our opinion this would be an erroneous conclusion, since the whole visual pathway is already optimized to contend with the optical limitations. Therefore, correction of the optical limitations is not able to improve the situation further and it is necessary to invoke neuronal processing (including photoreceptor accommodation, RF structure and size, the different neuronal processing pathways, etc.).

Furthermore, LCA is expected to be manifested not only adjacently to achromatic edges but also in many other spatial and chromatic configurations. For example, one would also expect LCA at iso-luminance chromatic edges and non-oriented edges (such as textures or dots on a uniform background). In such configurations, the visual image is clear, despite the fact that the “leakage” of short-wavelength colors is still expected to influence the chromatic appearance, and the postulated models are unable to provide compensation.

The strength of a computational model can be enhanced by showing its ability to predict additional phenomena. Evidence for the competence of our model comes from its ability to predict the enigmatic visual phenomenon of the large chromatic shifts by *S*-cone pattern (Shevell and Monnier, 2005; Figure 5).

Shevell and Monnier (2006) and Cao and Shevell (2005) suggested that the large color shifts are mediated by a spatially antagonist *S* + /*S*− cortical RF. The “*S*” term referred to the *S*-cone response normalized by the luminance. Cells with this type of response while not found in the retina have been identified in some neurons in V1 and V2 visual areas (Conway, 2001). Significantly, our model is based on retinal RFs (rather than cortical) (Hubel and Wiesel, 1968; de Monasterio, 1978; Derrington et al., 1984).

In addition, Shevell et al. also showed that the effect is more prominent with high-spatial frequency of the rings. We assume that this was the incentive to include spatially antagonist RFs in their qualitative model. We suggest, however, that an additional mechanism is recruited for low-frequency stimuli, i.e., simultaneous contrast mechanism (see Model, adaptation of the first order). Such a mechanism could originate from a retinal source (Spitzer and Barkan, 2005). This suggestion should be supported by additional experimental data, which should determine whether the effect originates from retinal vs. cortical mechanisms, as suggested previously (Cao and Shevell, 2005; Shevell and Monnier, 2006).

In summary, in this manuscript, we propose a model which explains how the visual system compensates for LCA. This compensatory mechanism can also explain additional visual phenomena, such as the large chromatic shifts by *S*-cone pattern, for which the underlying mechanism is still unknown. In addition, this mechanism can explain the necessity for two separate chromatic visual pathways, i.e., koniocellular and parvocellular pathways.

## Author Contributions

This is an original research done by YB under the supervision and parternship with HS.

## Conflict of Interest Statement

The authors declare that the research was conducted in the absence of any commercial or financial relationships that could be construed as a potential conflict of interest.

## Footnotes

^1^https://github.com/isetbio/.

^2^http://www.imageval.com/public/Products/ISET-SceneDatabase.html.
